# Alpha-hemolysin of uropathogenic *Escherichia coli* induces GM-CSF-mediated acute kidney injury

**DOI:** 10.1038/s41385-019-0225-6

**Published:** 2019-11-12

**Authors:** Changying Wang, Qianqian Li, Junqiang Lv, Xuan Sun, Yang Cao, Kaiyuan Yu, Chunhui Miao, Zhi-Song Zhang, Zhi Yao, Quan Wang

**Affiliations:** 1 Department of Immunology, Key Laboratory of Immune Microenvironment and Disease of the Educational Ministry of China, Tianjin Key Laboratory of Cellular and Molecular Immunology, School of Basic Medical Sciences, Tianjin Medical University, 300070 Tianjin, China; 2 Department of Clinical Laboratory, The Second Hospital of Tianjin Medical University, 300211 Tianjin, China; 3 State Key Laboratory of Medicinal Chemical Biology and College of Pharmacy, Collaborative Innovation Center for Biotherapy, and Tianjin Key Laboratory of Molecular Drug Research, Nankai University, 300350 Tianjin, China; 4 2011 Collaborative Innovation Center of Tianjin for Medical Epigenetics, Tianjin Medical University, 300070 Tianjin, China

## Abstract

Uropathogenic *Escherichia coli* (UPEC) is the leading cause of urinary tract infections (UTIs), inducing acute pyelonephritis and may result in permanent renal scarring and failure. Alpha-hemolysin (HlyA), a key UPEC toxin, causes serious tissue damage; however, the mechanism through which HlyA induces kidney injury remains unclear. In the present study, granulocyte-macrophage colony-stimulating factor (GM-CSF) secreted by renal epithelial cells was upregulated by HlyA in vitro and in vivo, which induced M1 macrophage accumulation in kidney, and ADAM10 was found involved in HlyA-induced GM-CSF. Macrophage elimination or GM-CSF neutralization protected against acute kidney injury in mice, and increased GM-CSF was detected in urine of patients infected by *hlyA*-positive UPEC. In addition, HlyA was found to promote UPEC invasion into renal epithelial cells by interacting with Nectin-2 in vitro. However, HlyA did not affect bacterial titers during acute kidney infections, and HlyA-induced invasion did not contribute to GM-CSF upregulation in vitro, which indicate that HlyA-induced GM-CSF is independent of bacteria invasion. The role of GM-CSF in HlyA-mediated kidney injury may lead to novel strategies to treat acute pyelonephritis.

## Introduction

Urinary tract infections (UTIs), which are one of the most common infectious diseases, affect >100 million people annually worldwide.^1^ UTIs, such as pyelonephritis, are the precursors of renal scarring and failure, especially in pediatric patients.^2,3^ Uropathogenic *Escherichia coli* (UPEC) is the main cause of UTIs, accounting for most community (~80–90%) and hospital acquired (~50%) infections.^1,4^

Virulence factors of UPEC that contribute to pathogenesis of UTIs mainly include fimbriae involved in adherence and invasion to host cells, toxins affecting host cells, and iron-acquisition systems for bacterial growth.^3,5^ Alpha-hemolysin of UPEC, HlyA, is cytotoxic to a wide range of cells and causes serious tissue damage during UTIs.^6^ The *hlyA* gene is located in the operon, including *hlyC*, *hlyA*, *hlyB*, and *hlyD*. HlyC is an acyltransferase that activates HlyA, and HlyB and HlyD are involved in HlyA secretion.^7^ HlyA is reported to induce kidney inflammation and injury,^5,8^ and a higher percentage of *hlyA*-positive strains are isolated from pyelonephritis patients ( > 70%) than from cystitis patients (31–48%), implying that HlyA is an important virulence factor in pyelonephritis.^7^ In vitro studies have shown that HlyA lyses cells by forming pores on cell membrane at high concentrations;^6,7^ HlyA disrupts cell adhesion, triggers urothelial cell death, and induces inflammatory cytokines from epithelial cells or monocytes via cell signaling pathways at low concentrations.^8–11^ For examples, HlyA promotes IL-6 and IL-8 secretion through Ca^2+^ oscillations in renal epithelial cells^11^ and triggers IL-1β release and cell death by activating NLRP3 inflammasome in epithelial cells and monocytes.^9,10^ In addition, HlyA has been reported to enhance exfoliation of bladder epithelial cells by inducing caspase-1/4-dependent inflammatory cells death in vivo.^9^ However, the mechanism by which HlyA causes kidney tissue damage during acute pyelonephritis remains unclear.

Granulocyte-macrophage colony-stimulating factor (GM-CSF) plays an important role in inflammation; however, the role of GM-CSF in kidney injury during acute pyelonephritis is unknown. GM-CSF was originally defined as a cytokine that promotes granulocytes and macrophages generation from bone marrow precursors.^12^ Recent reports indicate that GM-CSF is secreted in damaged tissues, which promotes monocyte infiltration into damaged tissues to sustain inflammation.^13,14^ Monocytes are recruited into inflamed tissues and mature into macrophages.^15^ GM-CSF has also been reported to promote M1 polarization, resulting in enhanced inflammation by M1 macrophage secreting inflammatory cytokines, such as IL-1β and TNFα.^14,16–20^ Some clinical trials that target GM-CSF or its receptor have been carried out for various inflammatory diseases, but not for pyelonephritis.^17^

Proteins on host cells that interact with bacterial virulence factors are important for bacterial pathogenicity. Nectins are cell adhesion molecules, highly conservative from humans to rodents, and include four members: Nectin-1, Nectin-2, Nectin-3, and Nectin-4.^21^ Most Nectins are membrane-associated proteins containing an extracellular region, a single transmembrane region, and a cytoplasmic region. As cell adhesion molecules, Nectins participate in the formation of adherens junctions and tight junctions in epithelial cells. Nectins also act as the entry receptors for viruses, and are involved in cell migration, proliferation, and polarization.^21,22^ ADAM10 has been reported to be the receptor of *Staphylococcus aureus* hemolysin (Hla) and is involved in cell death caused by hemolysins of other bacteria.^23,24^ The role of Nectins or ADAM10 in pathogenesis of HlyA has not been reported.

In the present study, HlyA was observed to induce GM-CSF-mediated M1 macrophage accumulation, which enhanced kidney injury. Macrophage elimination or GM-CSF neutralization greatly reduced HlyA-mediated kidney injury. ADAM10 in renal epithelial cells was involved in HlyA-induced GM-CSF secretion. Nectin-2 was identified to interact with HlyA and promote UPEC invasion into renal epithelial cells in vitro.

## Results

### HlyA promotes kidney injury and increases macrophage accumulation

**Fig. 1:**
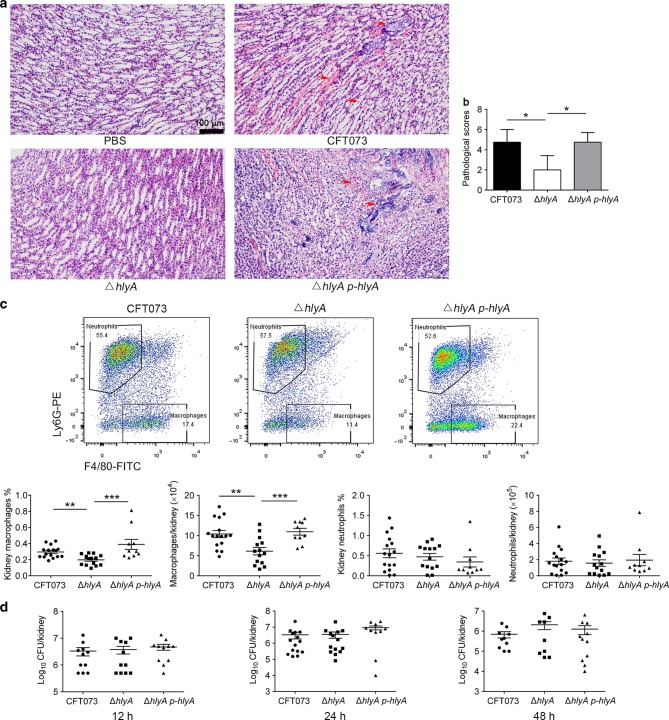
HlyA induces macrophage recruitment in an acute pyelonephritis mouse model. Female C57BL/6J mice were inoculated intraurethrally with 10^9^ CFU of CFT073, ∆*hlyA*, or ∆*hlyA p-hlyA* two times at a 3-h interval. **a** Representative images of H&E staining of kidney tissues at 24 hpi. The arrows indicate papillary necrosis, tubular casts, and serious hemorrhage. Scale bar, 100 μm. **b** Histological scores of kidney sections infected by CFT073, ∆*hlyA* or ∆*hlyA p-hlyA* at 24 hpi (*n* = 4). **c** Representative flow dot plots for CD11b^+^ cells in kidney and quantification of macrophages and neutrophils in kidney at 24 hpi (*n* = 10 to 16, three independent experiments). **d** Bacterial titers in kidney at 12, 24, or 48 hpi (*n* = 9 to 15, three independent experiments). Data are the mean ± SD (**b**) or ± SEM (**c**, **d**), one-way ANOVA (**b**) or nonparametric Mann–Whitney test (**c**, **d**). *P < 0.05, **P < 0.01, ***P < 0.001

To study the role of HlyA in kidney infection, UPEC strains CFT073, ∆*hlyA*, and ∆*hlyA p-hlyA* (the complemented strain), exhibiting similar growth rates (Supplementary Fig. S1a, b), were used to transurethrally infect female C57BL/6J mice separately. In kidney tissues infected with CFT073 or ∆*hlyA p-hlyA*, necrosis, tubular casts, and serious hemorrhage occurred in renal papillae (Fig. 1a, b and Supplementary Fig. S1c). Innate immune cells, including neutrophils and macrophages, in kidney tissues of mice infected with UPEC at 24 h post infection (hpi) were analyzed using flow cytometry. While there was no difference in infiltrated neutrophils, significantly more macrophages were detected in the CFT073 or ∆*hlyA p-hlyA* group compared with the ∆*hlyA* group (Fig. 1c and Supplementary Fig. S1d). We also examined bacterial titers in kidneys of C57BL/6J mice at 12, 24, and 48 hpi with CFT073, ∆*hlyA*, or ∆*hlyA p-hlyA*, and no statistically significant difference was observed (Fig. 1d). These results indicate that HlyA induces kidney injury and increases macrophages during acute kidney infections, which is independent of bacterial titers at the point in time we observe.

### HlyA induces GM-CSF secretion from renal epithelial cells

**Fig. 2:**
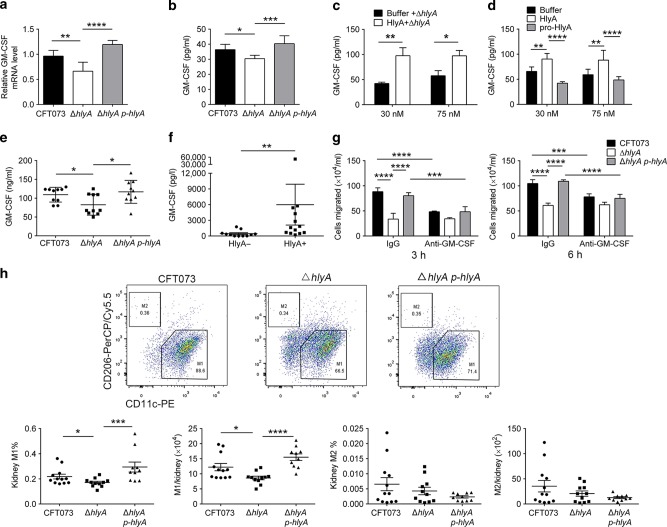
HlyA promotes GM-CSF secretion in renal epithelial cells during kidney infections. **a**, **b** GM-CSF mRNA (**a**) or secreted GM-CSF (**b**) in 786-O cells treated with CFT073, ∆*hlyA* or ∆*hlyA p-hlyA* (MOI 0.01) at 4 (**a**) or 6 (**b**) hpi (*n* = 3, three independent experiments each with two replicate wells). **c**, **d** Secreted GM-CSF in 786-O cells treated with recombinant HlyA (30 nM and 75 nM) or dialysis buffer for 12 h in combination with ∆*hlyA* (MOI 0.01) for 6 h (**c**) (*n* = 3, three independent experiments) or without ∆*hlyA* (*n* = 3, three independent experiments each with two replicate wells) (**d**). **e** GM-CSF in kidney tissues of female C57BL/6J mice infected with CFT073, ∆*hlyA* or ∆*hlyA p-hlyA* at 24 hpi (*n* = 10, three independent experiments). **f** GM-CSF in urine from patients with UTIs (*n* = 11 to 13). **g** Analysis of migrated THP-1 cells by cell supernatant from 786-O infected by CFT073, ∆*hlyA* or ∆*hlyA p-hlyA* at 3 and 6 hpi (*n* = 3, three independent experiments). **h** Representative flow dot plots of M1 or M2 in macrophages and quantification of M1 and M2 in kidney infected with CFT073, ∆*hlyA* or ∆*hlyA p-hlyA* at 24 hpi (*n* = 10 to 12, three independent experiments). Data are the mean ± SD (**a** to **e**, and **g**) or the mean ± SEM (**f** and **h**), one-way ANOVA (**a** to **d** and **g**) or nonparametric Mann–Whitney test (**e**, **f**, and **h**), *P < 0.05, **P < 0.01, ***P < 0.001, ****P < 0.0001

During kidney infections, UPEC strains interact with renal epithelial cells to mobilize the immune responses.^25–27^ Therefore, we hypothesized that HlyA induced chemokines by interacting with renal epithelial cells, which resulted in monocytes infiltration and increased macrophages. To determine the effect of HlyA on chemokines induction, CFT073 or ∆*hlyA* was used to treat the human renal epithelial cell line 786-O, and the messenger RNA (mRNA) levels of different kinds of chemokines were analyzed using quantitative reverse transcription PCR (qRT-PCR). The GM-CSF mRNA level was significantly higher in cells infected with CFT073 or ∆*hlyA p-hlyA* than in those infected with ∆*hlyA* (Fig. 2a and Supplementary Table S1). The secretion of GM-CSF by 786-O cells increased when the cells were infected with CFT073 or ∆*hlyA p-hlyA* compared with those infected with ∆*hlyA* (Fig. 2b). In order to exclude other effects caused by ∆*hlyA* mutant strain, ∆*hlyA* in addition with recombinant FLAG-tagged HlyA protein or dialysis buffer (control of recombinant FLAG-tagged HlyA protein) were used to treat 786-O cells, and more GM-CSF was detected in the recombinant HlyA group (Fig. 2c). We also examined the direct effect of recombinant FLAG-tagged HlyA to induce GM-CSF. Different doses of recombinant HlyA (that did not induce cell death at low concentrations), without any bacterial strain, also induced GM-CSF secretion; however, recombinant FLAG-tagged inactive HlyA protein (pro-HlyA) did not increase GM-CSF secretion (Fig. 2d and Supplementary Fig. S2a). To further validate HlyA’s effect on GM-CSF production in vivo, secreted GM-CSF was analyzed in kidney tissues infected with CFT073, *∆hlyA* or ∆*hlyA p-hlyA* at 24 hpi. A higher level of GM-CSF was detected in kidney infected with CFT073 or ∆*hlyA p-hlyA* than in that infected with *∆hlyA* (Fig. 2e). GM-CSF was reported to be elevated in urine of patients with UTIs in a recent study,^28^ and we found that GM-CSF level in urine of patients infected by *hlyA*-positive UPEC was obviously higher than that in patients infected by *hlyA*-negative UPEC (Fig. 2f and Supplementary Fig. S2b and Table S2). These results indicate that HlyA from UPEC induces GM-CSF secretion from renal epithelial cells during acute kidney infections.

### HlyA triggers monocyte migration and induces M1 macrophages in kidney during acute infections

GM-CSF induces monocyte migration and M1 differentiation.^14,17,19,29,30^ Given that HlyA promoted GM-CSF secretion by renal epithelial cells, we next examined the role of HlyA in monocyte migration and differentiation. Culture supernatant of 786-O cells infected with CFT073, ∆*hlyA* or ∆*hlyA p-hlyA* was used as the chemoattractant in Transwell migration assays, and the number of migrated monocytes was significantly higher for the CFT073 or ∆*hlyA p-hlyA* group compared with that for the ∆*hlyA* group (Fig. 2g). When anti-GM-CSF antibody was added in the supernatant, no difference of monocyte migration was observed for the CFT073, ∆*hlyA* or ∆*hlyA p-hlyA* group (Fig. 2g). In in vivo experiments, we found that, the levels of M1 macrophages were significantly higher in kidney tissues of mice infected with CFT073 or ∆*hlyA p-hlyA* compared with those infected with ∆*hlyA* at 24 hpi. Meanwhile, no difference was found for M2 macrophages (Fig. 2h). These results indicate that HlyA induces monocyte migration and increases M1 macrophages in kidney tissues during acute kidney infections with UPEC.

### Macrophage elimination or GM-CSF neutralization protects against acute kidney injury induced by HlyA

**Fig. 3:**
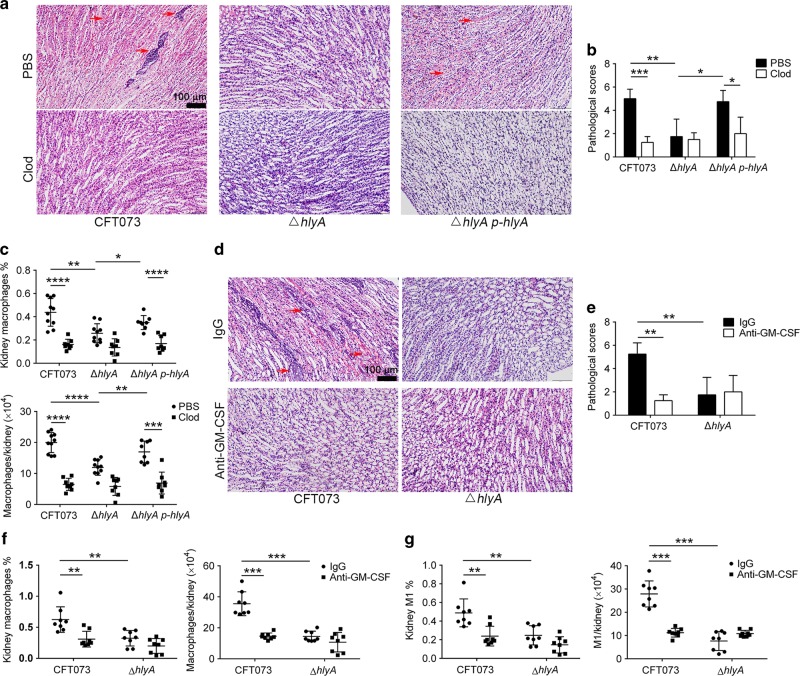
Macrophage elimination or GM-CSF neutralization attenuates kidney injury induced by HlyA. **a** H&E staining representative images of kidney tissues in mice treated with clodronate (Clod) liposomes or PBS liposomes and infected with CFT073, Δ*hlyA* or ∆*hlyA p-hlyA* at 24 hpi. The arrows indicate papillary necrosis, tubular casts, and serious hemorrhage. **b** Histological scores of kidney sections treated with Clod or PBS liposomes and infected by CFT073, ∆*hlyA* or ∆*hlyA p-hlyA* at 24 hpi (*n* = 4). **c** Percentages and numbers of macrophages in kidneys of mice treated with clodronate liposomes or PBS liposomes and infected with CFT073, Δ*hlyA* or ∆*hlyA p-hlyA* at 24 hpi (*n* = 8 to 10, two independent experiments). **d** H&E staining representative images of kidney tissues in mice treated with anti-GM-CSF antibody or control IgG and infected with CFT073 or Δ*hlyA* at 24 hpi. The arrows indicate papillary necrosis, and serious hemorrhage. Scale bar, 100 μm. **e** Histological scores of kidney sections treated with anti-GM-CSF antibody or control IgG and infected with CFT073 or ∆*hlyA* at 24 hpi (*n* = 4). **f**, **g** Analysis of macrophages in kidney treated with anti-GM-CSF antibody or control IgG and infected with CFT073 or Δ*hlyA* at 24 hpi (*n* = 8, two independent experiments). Data are the mean ± SD, one-way ANOVA (**b** and **e**) or nonparametric Mann–Whitney test (**c**, **f**, and **g**), *P < 0.5, **P < 0.01, ***P < 0.001, ****P < 0.0001

Although macrophages contribute to bacterial clearance, excessive amounts of macrophages result in exacerbated inflammation and tissue damage.^31^ To identify the role of increased macrophages in kidney injury caused by HlyA, clodronate (Clod) liposomes (to eliminate macrophages) or phosphate buffered saline (PBS) liposomes were injected intravenously into mice.^32,33^ Then the mice were infected with CFT073, ∆*hlyA* or ∆*hlyA p-hlyA* at 24 h post injection, respectively. Kidney injury caused by CFT073 or ∆*hlyA p-hlyA* at 24 hpi was obviously attenuated in mice treated with Clod liposomes compared with those treated with PBS liposomes, and no difference was found for mice infected with ∆*hlyA* (Fig. 3a, b). Meanwhile, macrophages in kidney tissues decreased markedly in the CFT073 or ∆*hlyA p-hlyA* group after treatment with Clod liposomes (Fig. 3c). Inflammatory cytokines in kidney at 24 hpi were also examined, and we found that IL-1β, TNF-α, IL-6, and MIP-2 were reduced in mice treated with Clod liposomes compared with those treated with PBS liposomes for the CFT073 or ∆*hlyA p-hlyA* group; however, no difference was found for the ∆*hlyA* group (Supplementary Fig. S3). Therefore, the increased macrophages triggered by HlyA play a role in kidney injury.

Given that HlyA induced GM-CSF secretion from renal epithelial cells, increased monocyte migration and M1 macrophages in kidney tissues, and GM-CSF is reported to promote monocyte migration and M1 differentiation,^14,17,19,29,30^ we hypothesized that HlyA induced M1 macrophage accumulation by increasing GM-CSF secretion. Therefore, anti-GM-CSF neutralization antibody or isotype control antibody was injected intravenously 1 h before CFT073 or ∆*hlyA* infection. After treatment with anti-GM-CSF antibody, kidney injury caused by CFT073 at 24 hpi decreased markedly compared with that in mice treated with isotype control, and no obvious difference was found in mice infected with ∆*hlyA* (Fig. 3d, e). Both total macrophages and M1 macrophages in kidney tissues were obviously lower in mice treated with anti-GM-CSF antibody (Fig. 3f, g). Thus, GM-CSF plays an important role in increased M1 macrophages and kidney injury by HlyA.

Taken together, these results imply that HlyA induces secretion of GM-CSF from renal epithelial cells, resulting in M1 macrophage accumulation in kidney, which exacerbates kidney injury.

### HlyA promotes UPEC invasion into renal epithelial cells

**Fig. 4:**
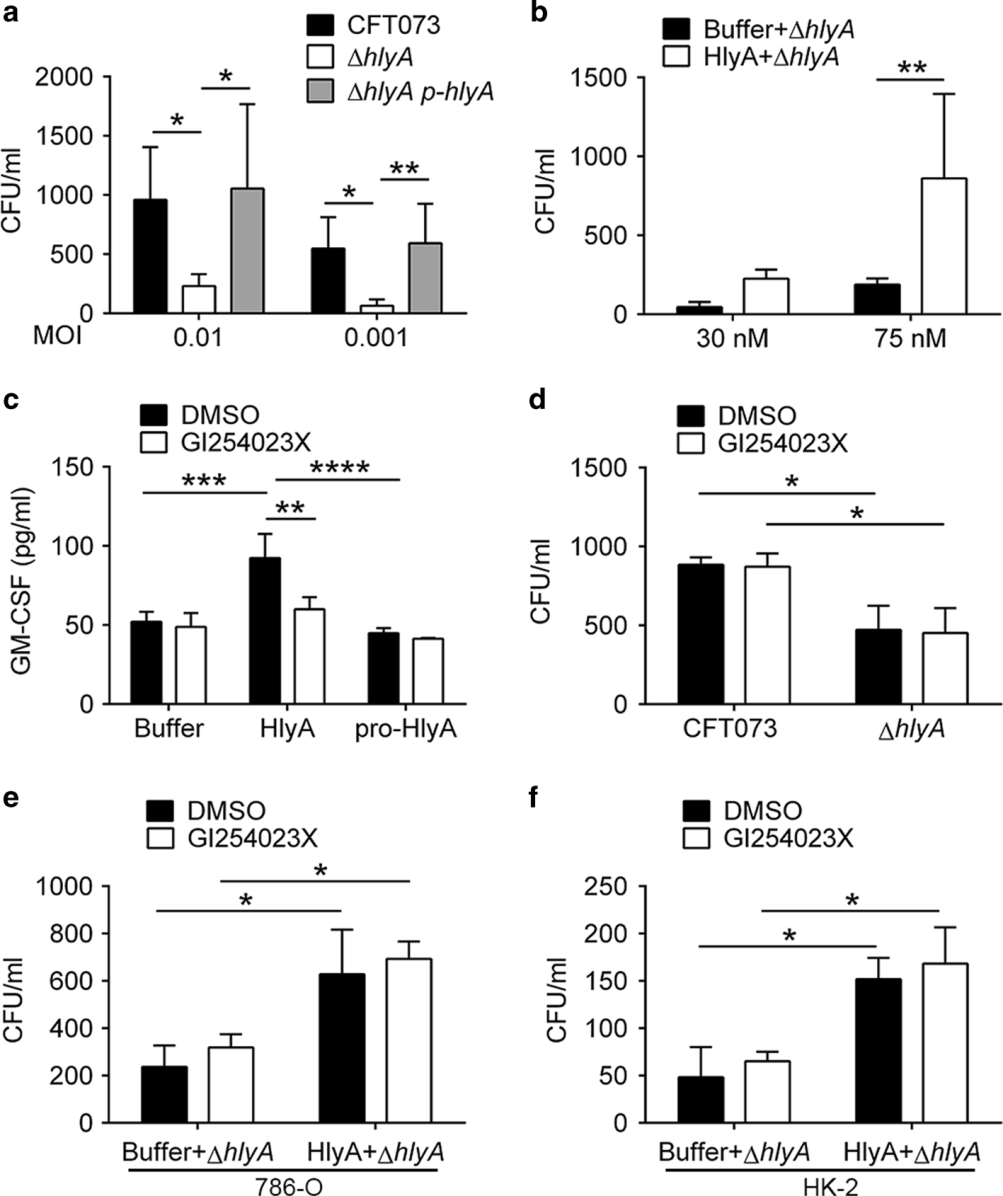
HlyA enhances bacterial invasion into renal epithelial cells. **a**, **b** Intracellular bacteria in 786-O cells infected with CFT073, Δ*hlyA* or ∆*hlyA p-hlyA* alone (**a**), or infected with ∆*hlyA* (MOI 0.01) in combination with dialysis buffer or HlyA (**b**) at 6 hpi were analyzed by killing extracellular bacteria using gentamicin (*n* = 3, three independent experiments each with two replicate wells). **c** Secreted GM-CSF of 786-O cells treated with dialysis buffer, HlyA or pro-HlyA (75 nM) for 12 h after incubation with DMSO or 20 μM GI254023X for 20 h (*n* = 3, three independent experiments). **d** Intracellular bacteria in 786-O cells infected with CFT073 or Δ*hlyA* (MOI 0.01) at 6 hpi after incubation with DMSO or GI254023X (20 μM) for 20 h (*n* = 3, three independent experiments). **e**, **f** Intracellular bacteria in 786-O (**e**) or HK-2 (**f**) cells infected with Δ*hlyA* (MOI 0.01) in combination with dialysis buffer or HlyA at 6 hpi after incubation with DMSO or GI254023X (20 μM) for 20 h (*n* = 3, three independent experiments). Data are the mean ± SD, one-way ANOVA, *P < 0.05, **P < 0.01, ***P < 0.001, ****P < 0.0001

Hemolysins of several strains, including *Listeria monocytogenes* and *S. aureus*, promote bacterial invasion into epithelial cells,^34,35^ and *hlyA*-positive *E. coli* strains showed higher invasion ability compared with *hlyA*-negative strains.^36^ Therefore, we hypothesized that HlyA would promote UPEC invasion into renal epithelial cells. In addition, lipopolysaccharides of Gram-negative bacteria can be recognized by TLR4/MD-2, and induces NF-κB activation,^37,38^ which is involved in GM-CSF transcription.^39^ Therefore, we hypothesized that HlyA induced GM-CSF partially by increasing UPEC invasion. Invasion of CFT073, ∆*hlyA* or ∆*hlyA p-hlyA* into 786-O cells was analyzed by killing extracellular bacteria using gentamicin, and ∆*hlyA* showed a lower invasion ability compared with that of CFT073 or ∆*hlyA p-hlyA* (Fig. 4a). The application of recombinant HlyA also improved invasion of ∆*hlyA* compared with dialysis buffer (Fig. 4b). To examine the effect of bacterial invasion in GM-CSF secretion, 786-O cells were treated with different doses of ∆*hlyA* (in order to exclude the direct effect of HlyA on GM-CSF secretion), and increased GM-CSF secretion was detected only for about 1000-fold increase of bacterial titers (Supplementary Fig. S4a, b). However, about three- to fivefold increase was observed for HlyA-induced UPEC invasion (Fig. 4a, b), the increased bacterial invasion by HlyA may not play a role in GM-CSF secretion.

We also wanted to know if ADAM10 (the receptor of *S. aureus* hemolysin Hla) was involved in HlyA-mediated GM-CSF secretion and UPEC invasion. After treatment using a specific ADAM10 inhibitor, GI254023X,^40,41^ the secretion of GM-CSF from 786-O cells induced by HlyA was decreased compared with those treated with DMSO (Fig. 4c). However, the ADAM10 inhibitor did not affect the UPEC invasion into 786-O or HK-2 cells induced by HlyA (Fig. 4d–f). In addition, the ADAM10 inhibitor did not affect HlyA cytopathic effect at low concentrations (Supplementary Fig. S4c)

These results suggest that HlyA promotes UPEC invasion in vitro, which does not contribute to GM-CSF secretion. ADAM10 plays a key role in HlyA-induced GM-CSF secretion, but not in UPEC invasion increased by HlyA.

### Nectin-2 is involved in increased UPEC invasion by HlyA

**Fig. 5:**
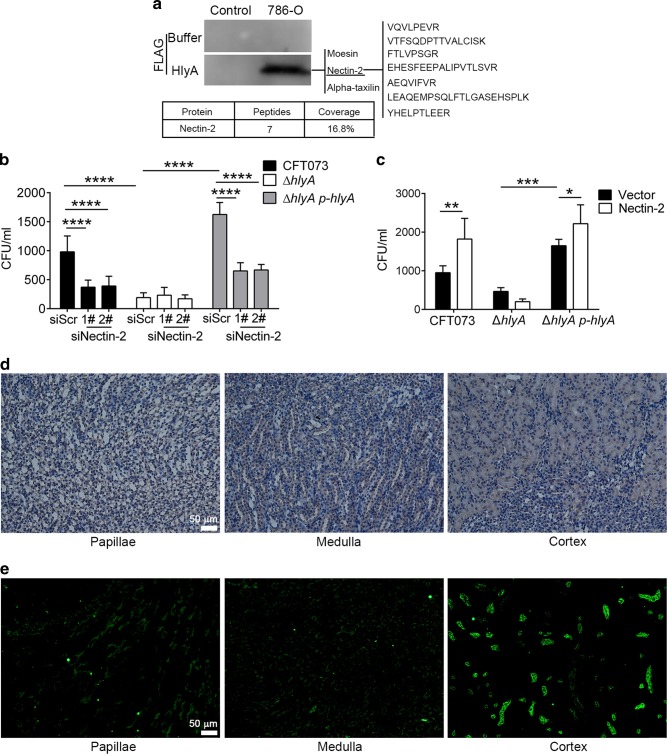
Nectin-2 is involved in HlyA-enhanced bacterial invasion into renal epithelial cells. **a** Analysis of 786-O cell membrane proteins binding to HlyA by far-western blotting and LC-MS/MS. The unique peptides detected and peptide coverage percentage of Nectin-2 are shown. **b** Intracellular bacteria in 786-O cells transfected with siRNAs targeting Nectin-2 or scramble non-targeting control siRNA infected with CFT073, Δ*hlyA* or ∆*hlyA p-hlyA* at 6 hpi (*n* = 3, three independent experiments). **c** Intracellular bacteria in 786-O cells transfected with vector or Nectin-2 infected with CFT073, Δ*hlyA* or ∆*hlyA p-hlyA* at 6 hpi (*n* = 3, three independent experiments). **d**, **e** Immunohistochemistry (**d**) and immunofluorescence (**e**) analysis of Nectin-2 expressed in renal papillary, medulla, cortex. Green, Nectin-2. Scale bar, 50 μm. Data are the mean ± SD, one-way ANOVA, *P < 0.05, **P < 0.01, ***P < 0.001, ****P < 0.0001

Based on the above results, we attempted to find the host protein interacting with HlyA and involved in UPEC invasion. Recombinant FLAG-tagged HlyA or dialysis buffer was incubated with 786-O membrane-associated proteins, and the specific band detected using far-western blotting was identified using liquid chromatography-tandem mass spectrometry (LC-MS/MS), which included three membrane-associated proteins (Moesin, Nectin-2 and Alpha-taxilin) (Fig. 5a). Nectin-2 knockdown in 786-O or HK-2 cells inhibited CFT073 or ∆*hlyA p-hlyA* but not ∆*hlyA* invasion (Fig. 5b and Supplementary Fig. S5a, b), whereas knockdown of Moesin or Alpha-taxilin did not show similar effects (Supplementary Fig. S5c). 786-O or HK-2 cells overexpressing Nectin-2 also showed enhanced CFT073 or ∆*hlyA p-hlyA* invasion, but not ∆*hlyA* invasion (Fig. 5c and Supplementary Fig. S5d, e). Nectin-2 knockdown or overexpression did not affect HlyA cytopathic effect (Supplementary Fig. S5f, g). No difference was observed for GM-CSF levels in Nectin-2 knockdown cells compared with those in control cells infected with CFT073 or ∆*hlyA*, implying that HlyA-mediated invasion does not contribute to GM-CSF increasement (Supplementary Fig. S5h). Nectin-2 expression was high in kidney tissues, as analyzed by immunohistochemistry and immunofluorescence assays (Fig. 5d, e). These results indicate that HlyA promotes UPEC invasion into renal epithelial cells through Nectin-2 in vitro; however, HlyA does not increase bacterial titers during acute kidney infections (Fig. 1d), which is speculated in the discussion section.

### HlyA interacts with Nectin-2 directly

**Fig. 6:**
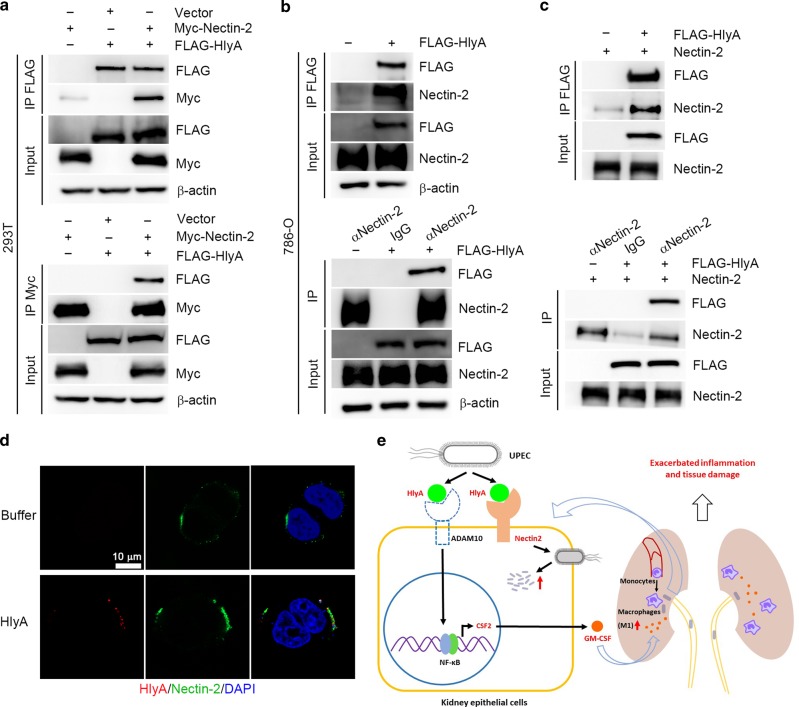
HlyA interacts with Nectin-2. **a** Co-immunoprecipitations analysis of the interaction between FLAG-tagged HlyA and Myc-tagged Nectin-2 expressed in 293T cells. **b** Co-immunoprecipitations analysis of the interaction between FLAG-tagged HlyA and Nectin-2 in 786-O cells. **c** Co-immunoprecipitations analysis of the interaction between purified FLAG-tagged HlyA and Nectin-2 protein. α, anti-. **d** HA-tagged HlyA and Myc-tagged Nectin-2 interactions in 293T cells analyzed by immunofluorescence assays. Scale bar, 10 μm. Blue, nucleus; red, HA-tagged HlyA; Green, Myc-tagged Nectin-2. **e** A proposed model that HlyA induces GM-CSF-mediated acute kidney injury

Nectin-2 is the receptor for herpes simplex viruses (HSV) 1 and 2 to enter into human epithelial cells.^42^ We hypothesized that Nectin-2 may interact with HlyA directly. 293T cells expressing Myc-tagged Nectin-2 were incubated with FLAG-tagged HlyA. Immunoprecipitation (IP) with anti-FLAG antibody followed by immunoblotting (IB) with anti-Myc antibody indicated that Nectin-2 was co-immunoprecipitated with recombinant HlyA. Reciprocally, IP using anti-Myc antibody followed by IB using anti-FLAG antibody showed that HlyA was co-immunoprecipitated with Nectin-2 (Fig. 6a). In addition, in 786-O cells treated with FLAG-tagged HlyA, IP with anti-FLAG or anti-Nectin-2 antibody followed by IB with anti-Nectin-2 or anti-FLAG antibody revealed that HlyA could bind to endogenous Nectin-2 (Fig. 6b). To examine if HlyA could bind to Nectin-2 directly, purified recombinant FLAG-tagged HlyA and Nectin-2 were used to perform in vitro pull-down experiments, which showed that purified FLAG-tagged HlyA and Nectin-2 proteins bound to each other directly (Fig. 6c). Immunofluorescence assays also showed that HA-tagged HlyA could bind to Myc-tagged Nectin-2 in 293T cells (Fig. 6d). Taken together, these results indicate that HlyA binds to Nectin-2 directly, and Nectin-2 plays a key role in increased UPEC invasion into renal epithelial cells by interacting with HlyA in vitro.

## Discussion

HlyA is an important virulence factor in the pathogenesis of pyelonephritis, and some studies found that HlyA-induced inflammatory cytokines, such as promoting IL-6, IL-8, or IL-1β secretion in vitro.^11^ The mechanism through which HlyA contribute to acute kidney injury has not been demonstrated. In the present study, we found that HlyA increased the mRNA and protein levels of GM-CSF in renal epithelial cells, which promoted M1 macrophage accumulation in kidney tissues. Using an acute pyelonephritis mouse model, we found that elimination of macrophages or neutralization of GM-CSF greatly attenuated HlyA-mediated acute kidney injury, which implies that increased GM-CSF and macrophages play important roles in kidney damage during acute pyelonephritis (Fig. 6e).

Hemolysin is an important virulence factor for many kinds of pathogenic bacteria. The most well studied hemolysin is Hla of *S.aureus.*^24^ ADAM10 was reported to be a receptor for Hla.^23^ The activation of ADAM10 leads to NF-κB signaling pathway activation,^43^ which upregulates GM-CSF transcription.^39^ In the present study, the ADAM10 inhibitor significantly reduced most of the GM-CSF induced by HlyA. Therefore, we speculated that HlyA increased GM-CSF through the ADAM10-NF-κB axis.

Two receptors for HlyA have been reported: CD11a/CD18 (LFA-1) and glycophorin. LFA-1 is expressed in B cells, T cells, neutrophils and monocytes, but not on epithelial cells and erythrocytes.^6,44^ Glycophorin is mainly expressed on erythrocyte surface.^45^ Nectin-2, also called CD112, is expressed in various tissue cells and hematopoietic cells. Nectin-2 acts as a cell adhesion molecule by forming a homo-trans-dimer or a hetero-trans-dimer.^21,46^ Nectins have also been reported as receptors for viruses to enter into host cells through promoting fusion between the viral envelope and the cellular plasma membrane or endocytosis.^47^ For example, Nectin-1 is the receptor for α-herpes viruses, Nectin-2 is the co-receptor for HSV-2 and HSV-1 mutant, and Nectin-4 is the receptor for the measles virus.^21^ Nectins were also reported to activate Cdc42 and Rac small GTP proteins, which promoted filopodia and lamellipodia formation.^48,49^ Activation of Rho GTPases promotes UPEC invasion into epithelial cells.^50,51^ In the present study, Nectin-2 was proved to interact with HlyA and was involved in increased UPEC invasion mediated by HlyA, implying that the direct interaction between HlyA and Nectin-2 may promote UPEC entry into renal cells by activation of small GTP proteins. In addition, whether HlyA increases UPEC adhesion to kidney epithelial cells through Nectin-2, which also enhance UPEC invasion, should be further studied. As the inhibitor or antibody for Nectin-2 is not commercially available, and Nectin-2 knockout mice exhibit male sterility (reported in Mouse Genome Informatics database, MGI database), it is hard to verify Nectin-2 effect on UPEC invasion in vivo. The role of Nectin-2 in kidney infections in vivo need to be further investigated.

Invasion of bacteria into epithelial cells is beneficial for their survival by escaping immune cell mediated clearance.^5^ Although hemolysins of some pathogens are involved in enhancing bacteria invasion, HlyA has not been proven to increase UPEC invasion.^36^ A previous study found that bacterial titers in kidney were not affected by HlyA.^5^ In our study, HlyA promoted UPEC invasion into renal epithelial cells, but did not affect bacterial titers in kidney tissues, which may be due to HlyA increased macrophage accumulation to clear bacteria, and it also explain why HlyA increased macrophage accumulation, but not decrease bacteria titers. However, it should be further studied.

GM-CSF is frequently expressed during inflammation to regulate myeloid cell numbers, and has little effect on embryonic development and hematopoiesis.^17,29,52^ In our study, GM-CSF neutralization using anti-GM-CSF antibody attenuated acute kidney injury caused by HlyA-positive UPEC strains, indicating that anti-GM-CSF therapy has the potential to treat serious acute pyelonephritis.

In this study, we elucidated the cellular and molecular mechanisms of acute kidney injury induced by HlyA, found that GM-CSF neutralization protects against HlyA-mediated kidney injury, and identified the host protein involved in HlyA-induced bacterial invasion. Antibiotics are usually used to treat UTIs, which can induce multidrug-resistant strains, and this study could lead to the development of alternative strategies to treat acute pyelonephritis.

## Methods

### Cell lines

The cell lines and their sources are as follows: 293T (ATCC CRL-11268), 786-O (ATCC CRL-1932), and HK-2 (ATCC CRL-2190). THP-1 cells were kindly provided by Stem Cell Bank, Chinese Academy of Sciences. The cells were grown in DMEM (for 293T and 786-O), MEM (for HK-2) or RPMI 1640 (THP-1) medium with 10% fetal bovine serum and 2 mM l-glutamine, at 37 °C in the presence of 5% CO2. Before infected by bacteria, treated by proteins or chemotaxis analysis, medium was changed to serum-free medium.

### Bacterial strains and plasmids

The bacterial strains and plasmids used in this study are listed in Supplementary Table S3. *E. coli* strains were cultured at 37 °C in Luria-Bertani (LB) medium under static conditions for 12 h with appropriate antibiotics when required, at the following concentrations: Kanamycin at 50 μg/ml, ampicillin at 100 μg/ml, and chloramphenicol at 15 μg/ml. The Δ*hlyA* strain was generated by the substitution of *hlyA* with a *cat* gene using λ-Red recombinase. To generate the ∆*hlyA p-hlyA* strain, the *hlyA* gene was amplified by PCR from the chromosome of UPEC strain CFT073 and ligated into pTRC99A at the KpnI and XbaI enzyme sites, and the plasmid was transformed into ∆*hlyA* by electroporation. The *hlyC* and *hlyA* genes from CFT073 or the complementary DNA (cDNA) encoding for Nectin-2 was amplified by PCR and cloned into pET-28a ( + ) to produce active FLAG or HA-tagged HlyA or Nectin-2 recombinant proteins. To produce inactive FLAG-tagged HlyA (pro-HlyA), the *hlyA* gene without *hlyC* from CFT073 was cloned into pET-28a ( + ) at XbaI and XhoI enzyme sites. Myc-tagged Nectin-2 was integrated into the pLenti-Hygro vector for transfection.

### HlyA, pro-HlyA, and human Nectin-2 recombinant protein expression and purification

Expression of recombinant HlyA or pro-HlyA was performed in *E. coli* BL21 (DE3), and expression of Nectin-2 was performed in Rosetta (DE3). Before induction with 100 μM IPTG, bacteria were cultivated at 37 °C until they reached an OD_600_ of 0.6 to 0.8. Cultured bacteria were collected by centrifugation (8000 × *g* for 5 min at 4 °C) after 12 h of induction at 16 °C in LB with IPTG. The bacteria were lysed with lysozyme and ultrasound and supernatant was centrifuged to remove particulates (18,000 × *g* for 30 min at 4 °C). The proteins were then purified using the Ni-NTA Purification System (GenScript, Nanjing, China). Proteins were eluted with 250 mM imidazole. Fractions with the HlyA fragments were pooled, dialyzed in 150 mM imidazole, 50 mM imidazole and twice with PBS. Subsequently, fractions containing the desired protein were concentrated to 500 μl using Amicon Ultra-15 Centrifugal Filter Units (Millipore, Burlington, MA, USA). The last dialysis buffer that had a similar ionic environment as the purified HlyA protein was used as the control for the purified protein in experiments. The final protein concentration was determined spectrophotometrically (Nanodrop-2000, Thermo Fisher Scientific, Waltham, MA, USA) using the BCA Protein Assay Kit (23225, Thermo Scientific).

### Mouse pyelonephritis model

All animal studies were reviewed and approved by the Animal Care and Use Committee at Tianjin Medical University, Tianjin, China. We made every effort to minimize animal suffering and to reduce the number of animals used. Female C57BL/6J mice, aged 6–8 weeks, were purchased from the Academy of Military Medical Science (Beijing, China). The acute pyelonephritis mouse model was established as previously described.^53^ The bacteria were cultured overnight in static LB medium at 37 °C. Cultured bacteria were pelleted by centrifugation (5000 × *g* for 5 min at 4 °C) and resuspended in PBS to obtain a density of 2 × 10^10^ CFU/ml. At a 3 h interval, anesthetized female C57BL/6J mice were inoculated intraurethrally with 50 μl UPEC strains (10^9^ CFU) twice.^54,55^ At 12, 24, and 48 hpi, mice were sacrificed, and their kidneys were aseptically removed and homogenized in 1 ml of PBS containing 0.025% Triton X-100, and then serially diluted for bacteria enumeration. At 24 hpi, the kidney tissues were also used for flow cytometry, histology, and pro-inflammatory cytokine analysis.

### Flow cytometry analysis

Single-cell suspensions were generated by digestion with 1.5 mg/ml collagenase IV (C5138, Sigma-Aldrich, St. Louis, MO, USA) and 100 ng/ml DNase I in PBS for 30 min at 37 °C under mild shaking. The digested cell suspensions were then filtered through a 70-μm cell strainer (352350, BD Biosciences, San Jose, CA, USA) to obtain single-cell suspensions. Fc receptors were blocked using CD16/32 (101319, Biolegend, San Diego, CA, USA) and the single-cell suspensions were then incubated with the following antibodies: anti-CD11b conjugated to APC (17-0112-82, Thermo Fisher Scientific), anti-Ly6G conjugated to PE (127608, Biolegend), anti-F4/80 conjugated to FITC (11-4801-82, Thermo Fisher Scientific), anti-CD11c conjugated to PE (127608, Biolegend), anti-CD206 conjugated to PerCP/Cy5.5 (141716, Biolegend). Cells were analyzed on a FACSCanto II Flow Cytometer (BD Biosciences) using the Flow Jo software (FlowJo, Ashland, OR, USA).

### H&E staining and immunohistochemistry

Kidneys were fixed in 10% phosphate-buffered formalin for at least 24 h. The fixed tissue was then embedded in paraffin and cut into 5-μm sections. The slides were stained with hematoxylin and eosin. Renal histopathological changes were assessed using a 6-point scale in which 0, 1, 2, and 3 indicated normal, mild, moderate, and severe histological lesions (pathological damage was mainly located within the medulla and the cortical-medullar junction); meanwhile 4, 5, and 6 indicated mild, moderate, and severe histological lesions (pathological damage was mainly located in more parts of the kidney). Histological examinations were analyzed by two persons who were blinded to experimental groups.^56,57^ For immunohistochemistry analysis, sections were stained with anti-Nectin-2 antibody (27171-I-AP, 1:200, Proteintech, Chicago, IL, USA). Images were acquired under a microscope (BX46, Olympus, Tokyo, Japan).

### Immunofluorescence analysis of tissues and cells

The kidneys were embedded in OCT compound with liquid nitrogen. Frozen blocks were cut into 5-µm sections, and air-dried at room temperature for 1 h, and fixed with cold acetone for 10 min. The frozen sections were then immediately submerged into methanol for 20 min and then methanol with 3% hydrogen peroxide for 10 min. The tissues were blocked with 5% bovine serum albumin (BSA) for 1 h, incubated with anti-F4/80 antibody (ab6640, Abcam, 1:200), anti-Ly6G antibody (ab210402, Abcam, 1:200), Nectin-2 antibody, (ab135246, Abcam, 1:200) in blocking buffer overnight at 4 °C when required. Slides were then washed five times with PBS, and incubated with Alexa Fluor 488/549-labeled secondary antibody (Proteintech, 1:200) for 1 h at room temperature. For nuclei visualization, tissue sections were counterstained with DAPI. Images were acquired under a fluorescent microscope (IX73, Olympus). The 293T cells transfected with pLenti-Hygro-Myc-Nectin-2 were grown on a Lab-Tek chambered coverglass and treated with 75 nM HlyA for 6 h, fixed with 4% paraformaldehyde for 15 min and subjected to immunofluorescence staining with anti-MYC-Tag antibody (66003-2-Ig, 1:25, Proteintech) and HA-Tag antibody (2367S, 1:200, CST) at 4 °C overnight. Alexa Fluor 488/594-labeled second antibody (Proteintech) were used by incubating at room temperature for 1 h. Cells were imaged using a confocal fluorescence microscope (FV1000-D, Olympus).

### Infection of kidney epithelial cells with UPEC strains

Human kidney epithelial cells (786-O or HK-2) were seeded in 24-well plates, 24 h before UPEC infections. For ADAM10 inhibition, cells were pre-incubated with the ADAM10 inhibitor GI254023X (Sigma-Aldrich) for 20 h before infections. Cells were infected with bacteria at the indicated multiplicity of infection (MOI) for 6 h or stimulated with the indicated concentrations of purified HlyA or pro-HlyA for 12 h. In some experiments, cells were treated with ∆*hlyA* (MOI 0.01) spiked with purified HlyA (75 nM) for 6 h. For invasion assay, after 6 h infection, the cells were washed five times with PBS and treated with 200 μg/ml of gentamicin for 1 h to kill extracellular bacteria. The cells were then washed two times with PBS and lysed with 500 μl of 0.2% triton X-100 in PBS and plated on LB agar plates to enumerate the intracellular bacteria.

### Enzyme-linked immunosorbent assay (ELISA)

GM-CSF levels in the supernatant from infected 786-O cells, HlyA/pro-HlyA-treated 786-O cells, or homogenized kidneys post infection were measured using an ELISA development kit (Neobioscience Technology Company, Shenzhen, China) according to the manufacturer’s instructions. The kidneys of mice were removed and homogenized in PBS containing 1% Triton X-100 and complete mini-EDTA-free protease inhibitor cocktail tablets (11697498001, Roche, Indianapolis, IN). Homogenates were then incubated on ice for 30 min and centrifuged at 10,000 × *g* for 10 min at 4 °C; supernatants were collected and used for ELISA assay of GM-CSF, IL-1β, TNF-α, IL-6, and MIP-2 according to the manufacturer’s instruction (Neobioscience Technology Company, Shenzhen, China).

### Cytotoxicity assays

Cell culture supernatants from 786-O cells treated by purified proteins or dialysis buffer for 12 h were collected, and detected for lactate dehydrogenase (LDH) using a CytoTox-96 Non-Radioactive Cytotoxicity Assay Kit (G1780, Promega, Madison, WI, USA).

### Urine samples from patients

Urine samples were collected from patients infected by UPEC strains underwent treatment at the Second Hospital of Tianjin Medical University, and the presence of *hlyA* gene in the UPEC strain isolated from urine of individual patient was determined by PCR (Supplementary Tables S2 and S4). Urine was concentrated to 200 μl using Amicon Ultra-15 Centrifugal Filter Units (UFC901024, Millipore), and then concentrated liquid was detected by ELISA development kit (Neobioscience Technology Company). The studies associated with patient samples were approved by the Ethics Committee of Tianjin Medical University, and the written informed consent was obtained from all patients.

### RNA extraction and qRT-PCR

786-O cells were infected with CFT073, ∆*hlyA* or ∆*hlyA p-hlyA* (MOI 0.01) for 4 h. RNA was extracted using a Total RNA Extraction Kit (Solarbio, Beijing, China) according to the manufacturer’s protocol and reverse-transcribed using the RevertAid First Strand cDNA Synthesis Kit (Thermo Fisher Scientific). qRT-PCR was performed using a FastStart Universal SYBR Green Master mix (Roche, Basel, Switzerland) on a 7900 Fast Real-Time PCR System (Roche). The PCR cycling conditions were 95 °C for 5 min, followed by 40 cycles of 95 °C for 20 s, 60 °C for 20 s, and 72 °C for 20 s. β-actin was used as the endogenous control and data were normalized based on the transcription level of β-actin in the wild-type and quantified using the comparative critical threshold cycle 2^–∆∆Ct^ method. The primers used are listed in Supplementary Table S4.

### Chemotaxis assays

Cell migration assays were performed using Transwell chambers (pore size 5 μm, Costar, Corning 3421, Corning, NY, USA). THP-1 cells (2 × 10^6^ in 200 μl) were re-suspended in serum-free RPMI 1640 medium and added to the upper chamber. Supernatant from infected 786-O cells was mixed with 1 μg/ml neutralizing antibody against human GM-CSF (502203, BVD2-23B6, Biolegend) or control IgG2a (400515, Rat IgG2a, Biolegend) and incubated for 30 min. Then, 600 μl of medium containing the supernatant was added to the lower chamber as the chemoattractant. After 3 h or 6 h of incubation at 37 °C in a 5% CO2 humidified atmosphere, the migrated cells in the lower chamber were counted.

### Clodronate liposomes and anti-GM-CSF antibody treatment

To eliminate macrophages, 200 µl of PBS or clodronate liposomes were administered to mice intravenously 24 h before infection.^32,33^ To neutralize GM-CSF, neutralizing antibody against GM-CSF (505408, MP1-22E9, Biolegend, 250 µg) or control IgG2a (400533, Rat IgG2a, Biolegend, 250 µg) was injected intravenously into mice 1 h before infection.^33,58^

### Antibodies and western blotting

Antibodies were obtained from the following companies: monoclonal anti-FLAG antibody (F1804, Sigma-Aldrich), anti-MYC-Tag antibody (66004-I-Ig, Proteintech, Chicago, IL, USA), and anti-Nectin-2 antibody (ab135246, Abcam, Cambridge, UK). Whole-cell lysates were prepared using RIPA lysis buffer (Millipore), with complete protease inhibitors (Roche, Basel, Switzerland). The BCA Protein Assay Kit (Thermo Fisher) was used to determine the protein concentration. The HRP-conjugated anti-rabbit IgG (1:10000, Sigma-Aldrich) or anti-mouse IgG (1:10000, Sigma-Aldrich) were used to reveal antibody binding. Immunoreactive complexes were detected using Immobilon Western Chemiluminescent HRP Substrate (Millipore) and exposed to a GE Amersham Imager 600 machine.

### Far-western blotting and LC-MS/MS analysis

The far-western blotting protocol was performed as previously described.^59^ 786-O cells were washed twice with ice-cold PBS, and the cell membrane proteins were isolated using a Membrane and Cytosol Protein Extraction Kit (Beyotime Biotechnology, Shanghai, China) according to the manufacturer’s protocol. Soluble membrane-associated proteins were analyzed using sodium dodecyl sulfate–polyacrylamide gel electrophoresis on 10% gels. The proteins were then transferred to polyvinylidene fluoride (PVDF) membrane (Merck Millipore, Darmstadt, Germany). The transferred proteins were renatured using AC buffer by gradually reducing the guanidine-HCl concentration.^59^ Then, the membrane was blocked with 5% skimmed milk in the TBST buffer for 1 h. Thereafter the membrane was incubated with 30 μg/ml purified FLAG-tagged HlyA or dialysis buffer overnight at 4 °C. After washing, the membrane was incubated with 1:1000 diluted anti-FLAG antibody (Sigma-Aldrich) overnight at 4 °C in 5% skimmed milk in the TBST buffer. Then the membrane was washed thoroughly and incubated with HRP-conjugated anti-mouse IgG (1:10000, Sigma-Aldrich)

The differential bands between the dialysis buffer group and the FLAG-tagged HlyA group was identified using LC-MS/MS, performed using a nanoLC-LTQ-Orbitrap XL mass spectrometer (Thermo, San Jose, CA, USA) coupled with an Eksigent nano LC 1D plus HPLC system in Majorbio (Shanghai, China). Tryptic peptides were fully enzymatically digested and ionized using nano electrospray ionization.^33^ Data were analyzed using a full-scan mass spectrum (300 to 1800 m/z). Finally, Proteome Discoverer (version 1.4.0.288, Thermo Scientific) was used to analyze the MS data.

### RNA interference and Nectin-2 overexpression

Small-interfering RNAs (siRNAs) for the targeted genes and a scrambled control siRNA (siScr) were synthesized by GenePharma (Shanghai, China). The siRNAs were transfected into 786-O or HK-2 cells using Lipofectamine 3000 (Invitrogen). pLenti-Hygro-Myc-Nectin-2 was transfected into 786-O or HK-2 cells using Lipofectamine 3000 (Invitrogen) to overexpress Nectin-2. Forty-eight hours post transfection, the cells were analyzed for protein expression using western blotting. The sequences of the siRNAs are listed in Suplementary Table S4.

### Immunoprecipitation

293T cells were transfected with the pLenti-Hygro vector or pLenti-Hygro-Myc-Nectin-2, and then cultured for 48 h. The cells were then incubated with FLAG-tagged HlyA (75 nM) for 6 h after transfection and were freshly lysed in lysis buffer (50 mM Tris-HCl (pH 7.4), 1 % NP-40, 0.2 mM EDTA, 150 mM NaCl) for western blotting or immunoprecipitation (IP) assays. 786-O cells were incubated with FLAG-tagged HlyA (75 nM) or dialysis buffer for 6 h and then lysed using lysis buffer for protein IP assay. Cell supernatants were incubated with anti-FLAG M2 beads (A2220, Sigma-Aldrich) or anti-Myc M2 beads (A7470, Sigma-Aldrich) for 12 h at 4 °C for FLAG-tagged or Myc-tagged protein IP. For Nectin-2 protein IP, cell supernatants were incubated with anti-Nectin-2 antibody (ab135246, Abcam) for 12 h at 4 °C, and then incubated with Protein A/G agarose (20241, Thermo Fisher) for 2 h at 4 °C. Normal Rabbit IgG (2729S, CST) was used as the control. After incubation, the precipitates were collected via centrifugation, washed five times with the lysis buffer, and analyzed by immunoblotting using monoclonal anti-FLAG antibody, anti-MYC-Tag antibody, or anti-Nectin-2 antibody.

Bacterial expressed, purified recombinant FLAG-tagged HlyA (1 µg) was incubated with 1 μg of bacterial expressed purified recombinant Nectin-2 in binding buffer (20 mM Tris-HCl (pH 7.4), 0.1 % Triton-X 100, 100 mM NaCl, 20% glycerin,1% BSA) for 12 h. The complexes were then subjected to FLAG-tagged protein IP or Nectin-2 protein IP. Finally, the complexed proteins were analyzed using immunoblotting.

### Statistical analysis

The statistical significance of the differences between groups was tested using analysis of variance (ANOVA) analysis. The nonparametric Mann–Whitney test was used to calculate the statistical significance in the in vivo experiments.

## Supplementary information

Supplementary Materials
