# Supplementary material for: Alpha-hemolysin of uropathogenic Escherichia coli induces GM-CSF-mediated acute kidney injury
Source: Mucosal Immunol. 2019 Nov 12;13(1):22–33. doi: 10.1038/s41385-019-0225-6 (PMC6914670; doi:10.1038/s41385-019-0225-6)
Supplement: Supplementary file 1 — Supplementary Materials [file 41385_2019_225_MOESM1_ESM.docx]

Supplementary Materials

Alpha-hemolysin of uropathogenic *Escherichia coli* induces GM-CSF-mediated acute kidney injury

Changying Wang, Qianqian Li, Junqiang Lv, Xuan Sun, Yang Cao, Kaiyuan Yu, Chunhui Miao, Zhi-Song Zhang, Zhi Yao, Quan Wang

**Corresponding Author:**

Quan Wang

Email: wangquan@tmu.edu.cn

Zhi Yao

Email: yaozhi@tmu.edu.cn

**This file includes:**

Figure legends

Figs. S1 to S5

Tables S1 to S4

Figure legends

Fig. S1. The **hemolytic activity and growth curve of CFT073, ∆*hlyA* or ∆*hlyA p-hlyA*.** (a) CFT073, ∆*hlyA* or ∆*hlyA p-hlyA* was streaked on blood plates, incubated overnight at 37 °C. (b) Growth curve of CFT073 or ∆*hlyA* in LB. (c) Kidneys from C57BL/6 mice infected with CFT073, ∆*hlyA* or ∆*hlyA p-hlyA* at 24 hpi. (d) Immunofluorescence analysis of macrophages and neutrophils in kidney at 24 hpi. Scale bar, 50 μm. Blue, nucleus; red, Ly6G; Green, F4/80 (n = 3, three independent experiments each with two fields). Data are the mean ± SD, non-parametric Mann-Whitney test, **P < 0.01.

Fig. S2. **Detection of *hlyA* in UPEC strains isolated from urine of patients.** (a) LDH assays of 786-O cells treated with 75, 150, 300 or 450 nM of recombinant HlyA or pro-HlyA for 12 h (n = 3, three independent experiments). (b) *hlyA* of *E. coli* isolated from urine of patients were amplified by PCR. Data are the mean ± SD, one-way ANOVA, ****P < 0.0001.

Fig. S3. Inflammatory cytokines in kidney affected by HlyA-induced macrophages accumulation. Mice were treated intravenously with clodronate (Clod) liposomes or PBS liposomes for 24 hours, and subsequently infected with CFT073, ∆*hlyA* or ∆*hlyA p-hlyA*. Intrarenal IL-1β, TNF-α, IL-6 and MIP-2 levels are evaluated by ELISA at 24 hpi (n = 3). Data are the mean ± SD, one-way ANOVA, *P < 0.05, **P < 0.01, ***P<0.001, ****P <0.0001.

Fig. S4. Effect of UPEC invasion on GM-CSF secretion in 786-O cells. (a) Intracellular bacteria in 786-O cells infected with Δ*hlyA* at 6 hpi (n = 3, three independent experiments). (b) Secreted GM-CSF in 786-O cells infected with Δ*hlyA* at 6 hpi (n = 3, three independent experiments). (c) LDH assays of 786-O cells pretreated with the ADAM10 inhibitor GI254023X (20 μM) or DMSO for 20 h, and subsequently treated with buffer, pro-HlyA or HlyA (75 nM). Supernatants were collected after treatment with the protein for 12 h and analyzed (n = 3, three independent experiments). Data are the mean ± SD, one-way ANOVA, *P < 0.05, ***P<0.001, ****P <0.0001.

Fig. S5. **Moesin and Alpha-taxilin are not involved in** HlyA enhanced **bacterial invasion.** (a) Western blotting analysis of Nectin-2 in 786-O cells transfected with siRNAs targeting Nectin-2 or scramble non-targeting control siRNA for 48 h. (b) Intracellular bacteria in HK-2 cells transfected with siRNAs targeting Nectin-2 or scramble non-targeting control siRNA infected with CFT073 or ∆*hlyA* (MOI 0.01) at 6 hpi (n = 3, three independent experiments). (c) Intracellular bacteria in 786-O cells transfected with siRNAs targeting Moesin or Alpha-taxilin or scramble non-targeting control siRNA infected with CFT073 or Δ*hlyA* (MOI 0.01) at 6 hpi (n = 3, three combined independent experiments). (d) Western blotting analysis of Nectin-2 in 786-O cells transfected with vector or Nectin-2 for 48 h. (e) Intracellular bacteria in HK-2 cells transfected with vector or Nectin-2 infected with CFT073 or Δ*hlyA* (MOI 0.01) at 6 hpi (n = 3, three independent experiments). (f-g) LDH assays of 786-O cells transfected with siRNAs targeting Nectin-2 (f) or overexpressing Nectin-2 (g) for 48 h, and subsequently treated with buffer, pro-HlyA or HlyA (75 nM). After 12 h of treatment, supernatants were collected and analyzed. (h) GM-CSF secretion of 786-O cells transfected with siRNAs targeting Nectin-2 or scramble non-targeting control siRNA and infected with CFT073 or Δ*hlyA* (MOI 0.01) at 6 hpi (n = 3, three combined independent experiments). Data are mean ± SD, one-way ANOVA, *P<0.05, **P <0.01, ***P <0.001.





**Fig. S1**


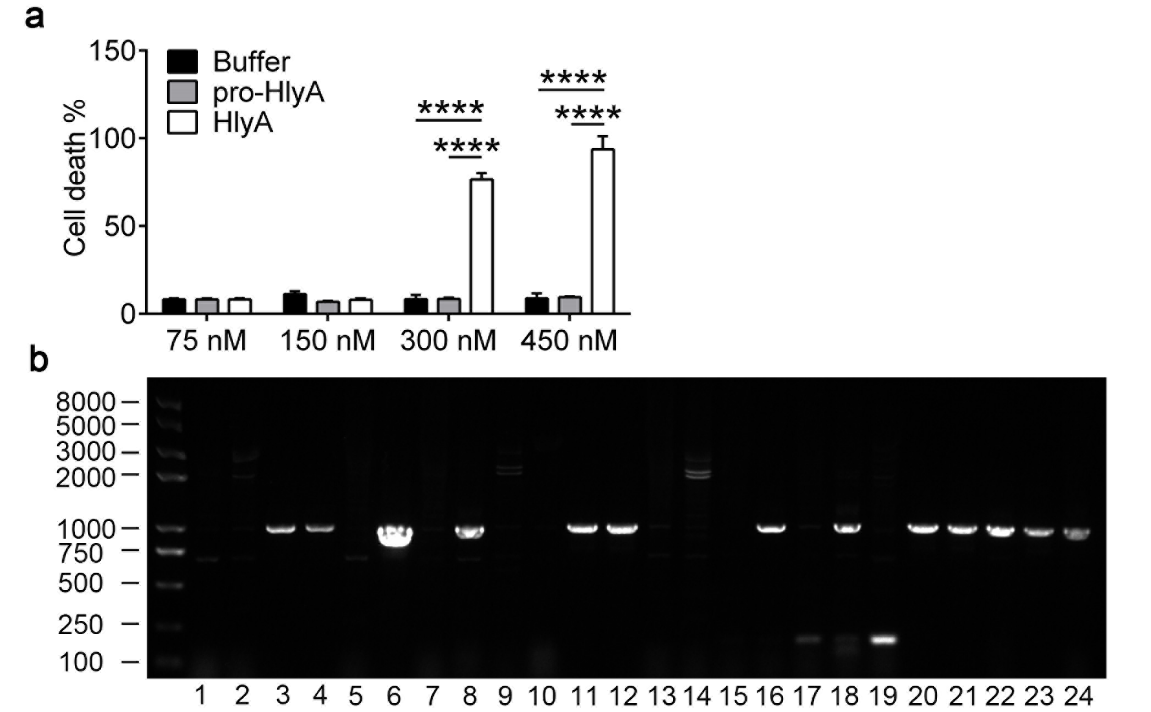


**Fig. S2**


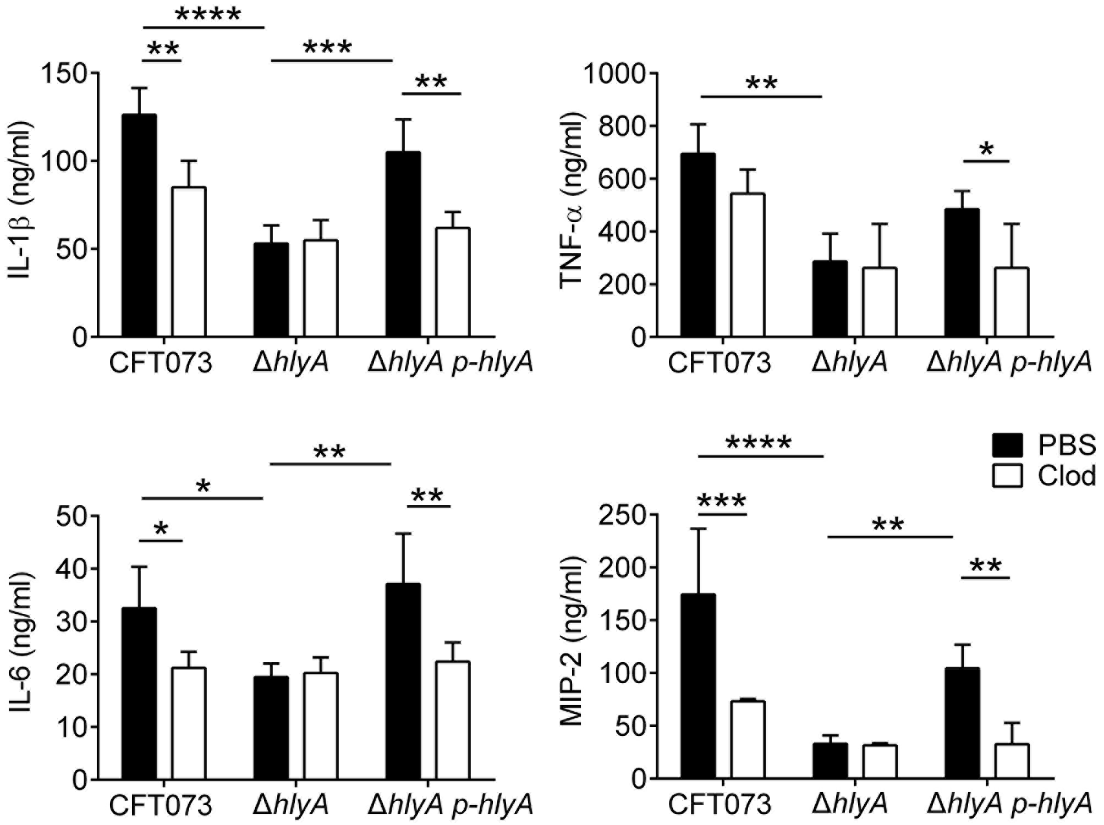


**Fig. S3**





**Fig. S4**





**Fig. S5**

Table S1. Relative **cytokine mRNA levels in 786-O cells infected with CFT073 or ∆*hlyA***

| **Gene** | **Relative mRNA level of**  **△*hlyA* /CFT073** | ***P* value** |
| --- | --- | --- |
| IL-1α | 1.25 | 0.48 |
| IL-1β | 0.93 | 0.54 |
| IL-8 | 0.86 | 0.16 |
| CCL2 | 0.99 | 0.93 |
| CCL5 | 1.1 | 0.49 |
| CCL8 | 1.13 | 0.69 |
| CCL11 | 1.07 | 0.89 |
| CCL20 | 0.81 | 0.18 |
| CXCL13 | 0.72 | 0.39 |
| CXCL14 | 1.77 | 0.29 |
| CX3CL1 | 1.23 | 0.35 |
| IFN-γ | 1.13 | 0.77 |
| MIF | 0.81 | 0.07 |
| M-CSF | 0.86 | 0.58 |
| GM-CSF | 0.68 | **0.0057** |

Note: n=3, three combined independent experiments, Student’s *t*-test.

**Table S2. Patient information in this study**

| Patient | Age(year) | Sex | Uropathogen | *hlyA* |
| --- | --- | --- | --- | --- |
| 1 | 68 | female | *E. coli* | ̶ |
| 2 | 64 | male | *E. coli* | ̶ |
| 3 | 71 | male | *E. coli* | + |
| 4 | 67 | male | *E. coli* | + |
| 5 | 76 | female | *E. coli* | ̶ |
| 6 | 82 | male | *E. coli* | + |
| 7 | 60 | male | *E. coli* | ̶ |
| 8 | 74 | female | *E. coli* | + |
| 9 | 83 | female | *E. coli* | ̶ |
| 10 | 60 | female | *E. coli* | ̶ |
| 11 | 83 | male | *E. coli* | + |
| 12 | 63 | male | *E. coli* | + |
| 13 | 30 | female | *E. coli* | ̶ |
| 14 | 66 | female | *E. coli* | ̶ |
| 15 | 61 | female | *E. coli* | ̶ |
| 16 | 57 | male | *E. coli* | + |
| 17 | 94 | male | *E. coli* | ̶ |
| 18 | 90 | female | *E. coli* | + |
| 19 | 64 | female | *E. coli* | ̶ |
| 20 | 67 | male | *E. coli* | + |
| 21 | 89 | female | *E. coli* | + |
| 22 | 49 | female | *E. coli* | + |
| 23 | 60 | male | *E. coli* | + |
| 24 | 52 | male | *E. coli* | + |

+, positive; ̶ , negative.

**Table S3. Strains and plasmids in this study**

| **Strain or plasmid** | **Description** | **Source** |
| --- | --- | --- |
| Bacterial strain |  |  |
| CFT073  Δ*hlyA*  Δ*hlyA* *p-hlyA* | Uropathogenic *E. coli* strain  *hlyA* deletion strain derived from CFT073  *hlyA* complemented strain derived from CFT073 | ATCC  This work  This work |
| *E.coli* BL21 | F^-^ *ompT hsdS_B_* ( *r_B_^-^ m_B_^-^*) *gal* *dcm* (DE3) | Novagen, Merck-Millipoe |
| Rosetta | F^-^ *ompT* *hsdS_B_ (r_B_^-^ m_B_^-^*) *gal dcm* (DE3) pRARE^2^(Cam^R^) | ATCC |
| *E. coli* DH5α | F^-^ *φ*80*lacZ* M15 *endA recA1* *hsdR* (r*_k_*^-^ m*_k_*^-^) *supE44* *thi-1* gyrA96 relA1_(*lacZYA-argF*) U169 | Beijing Dingguo Biotechmology Development Center, Beijing, China |
| Plasmid |  |  |
| PET-28a(+) | T7 expression vector, Kan^R^ | Novagen, Merck-Millipoe |
| pLenti-Hygro | Cloning vector, Amp^R^ | Novagen, Invitrogen |
| pTRC99A | Trc expression vector, Amp^R^ | Novagen, Invitrogen |
| pQW0601 | pET28a+ containg *hlyC* and C-terminal 6 x histidine and HA-tagged *hlyA* from CFT073 | This work |
| pQW0602 | pTRC99A containing *hlyA* from CFT073 | This work |
| pQW0604 | pET28a+ containg *hlyC* and C-terminal 6 x histidine and FLAG-tagged *hlyA* from UPEC | This work |
| pQW0606 | pET28a+ containg C-terminal 6 x histidine-tagged Nectin-2 | This work |
| pQW0607 | pLenti-Hygro containg 6 x Myc-tag | This work |
| pQW0608 | pLenti-Hygro containg 6 x Myc-tagged Nectin-2 | This work |
| pQW0619 | pET28a+ containg C-terminal 6 x histidine and FLAG tagged *hlyA* from UPEC | This work |

**Table S4. Primers and siRNAs used in this study**

| **Primer** | **Sequence (5’-3’)** | **Description** |
| --- | --- | --- |
| WQ0824 | CTAGTCTAGATTGGTTTGCTTTTTTTTACCTG | For cloning *hlyCA* into pET28a (+) to purify HA-tagged or FLAG-tagged HlyA |
| WQ0825 | CCGCTCGAGTTAGTGGTGGTGGTGGTGGTGAGCGTAATCTGGAACATCGTATGGGTACATTGCTGATGCTGTCAAAGT | For cloning *hlyCA* into pET28a (+) to purify HA-tagged HlyA |
| WQ 1244 | CCGCTCGAGTTAGTGATGATGATGATGATGCTTATCGTCGTCATCCTTGTAATCTGCTGATGCTGTCAAAGT | For cloning *hlyCA* or *hlyA* into pET28a (+) to purify FLAG-tagged HlyA or inactive HlyA |
| WQ 1860  WQ 0826  WQ 0827  WQ 1791 | CTAGTCTAGAATGCCAACAATAACCACTG  CGGGGTACCATGCCAACAATAACCACTG  CTAGTCTAGATTATGCTGATGCTGTCAAAG  TGCTGCCGGTTTAATTGCTTC | For cloning *hlyA* into pET28a (+) to purify FLAG-tagged inactive HlyA  For cloning *hlyA* into pTRC99A  For cloning *hlyA* into pTRC99A  For detecting *hlyA* in *E. coli* |
| WQ 1792 | GGCTGAGCCGGCAGATAAAA | For detecting *hlyA* in *E. coli* |
| WQ 1630 | CGCGGATCCATGGCCCGGGCCGCTGCCCTC | For cloning Nectin-2 into pET28a(+) |
| WQ 1631 | CCGCTCGAGCACATACATGGCCCGGGACATGAC | For cloning Nectin-2 into pET28a(+) |
| WQ1545 | CGGCTCGAGATGGCCCGGGCCGCTGCCCTC | For cloning Nectin-2 into pLenti-Hygro |
| WQ1546 | CGCGGATCCTCACACATACATGGCCCGGGACATGAC | For cloning Nectin-2 into pLenti-Hygro |
| WQ1269 | AGATGCCTGAGATACCCAAAACC | For human IL-1α qRT-PCR |
| WQ1270 | CCAAGCACACCCAGTAGTCT | For human IL-1α qRT-PCR |
| WQ1271 | GAGCTCGCCAGTGAAATGAT | For human IL-1β qRT-PCR |
| WQ1272 | CCTGAAGCCCTTGCTGTAGT | For human IL-1β qRT-PCR |
| WQ1277 | ACTGAGAGTGATTGAGAGTGGAC | For human IL-8 qRT-PCR |
| WQ1278 | AACCCTCTGCACCCAGTTTTC | For human IL-8 qRT-PCR |
| WQ1285 | CCTTCATTCCCCAAGGGCTC | For human CCL2 qRT-PCR |
| WQ1286 | GGTTTGCTTGTCCAGGTGGT | For human CCL2 qRT-PCR |
| WQ1289 | TCATTGCTACTGCCCTCTGC | For human CCL5 qRT-PCR |
| WQ1290 | TCGGGTGACAAAGACGACTG | For human CCL5 qRT-PCR |
| WQ1293 | TCATGCTGAAGCTCACACCC | For human CCL8 qRT-PCR |
| WQ1294 | GTCCCTGAGGGCTGAAAGTG | For human CCL8 qRT-PCR |
| WQ1295 | CCCCTTCAGCGACTAGAGAG | For human CCL11 qRT-PCR |
| WQ1296 | TCTTGGGGTCGGCACAGAT | For human CCL11 qRT-PCR |
| WQ1305 | GCGGCGAATCAGAAGCAAGC | For human CCL20 qRT-PCR |
| WQ1306 | TTTGGATTTGCGCACACAGAC | For human CCL20 qRT-PCR |
| WQ1309 | GCTTGAGGTGTAGATGTGTCC | For human CXCL13 qRT-PCR |
| WQ1310 | CCCACGGGGCAAGATTTGAA | For human CXCL13 qRT-PCR |
| WQ1311 | CCAAGATCCTGTGATGGCGA | For human CXCL14 qRT-PCR |
| WQ1312 | GCTGACAGTGTTGGGAACCT | For human CXCL14 qRT-PCR |
| WQ1313 | ACCACGGTGTGACGAAATG | For human CX3CL1 qRT-PCR |
| WQ1314 | TGTTGATAGTGGATGAGCAAAGC | For human CX3CL1 qRT-PCR |
| WQ1315 | TGTCGCCAGCAGCTAAAACA | For human IFN-γ qRT-PCR |
| WQ1315 | TGCAGGCAGGACAACCATTA | For human IFN-γ qRT-PCR |
| WQ1317 | CCGAGAAGTCAGGCACGTAG | For human MIF qRT-PCR |
| WQ1318 | ACATCGGCATGATGGCAGAA | For human MIF qRT-PCR |
| WQ0979 | AATGTTTGACCTCCAGGAGCC | For human GM-CSF qRT-PCR |
| WQ0998 | TCTGGGTTGCACAGGAAGTTT | For human GM-CSF qRT-PCR |
| WQ0999 | AAAGTTTGCCTGGGTCCTCT | For human M-CSF qRT-PCR |
| WQ1002 | AGACCAACAACAGCAGGGAG | For human M-CSF qRT-PCR |
| WQ1319 | AATGAGCTGCGTGTGGCT | For human β-actin qRT-PCR |
| WQ1320 | TAGCACAGCCTGGATAGCAA | For human β-actin qRT-PCR |
| 1# siNectin-2 | GACGAGGGCAACUACACUUTT | siRNA for Nectin-2 |
| 2# siNectin-2 | GCAUGAGAGCUUCGAGGAATT | siRNA for Nectin-2 |
| siMoesin | AGAUCGAGGAACAGACUAATT | siRNA for Moesin |
| siAlpha-taxilin | GCGAGGAGCAUAUCGACAATT | siRNA for Alpha-taxilin |
